# CLINICIAN AWARENESS, ATTITUDES, AND INTEREST IN BLOOD TESTS FOR ALZHEIMER’S DISEASE

**DOI:** 10.1093/geroni/igae098.3938

**Published:** 2024-12-31

**Authors:** Karen Tracy, Julie Wood, Ben Tiede, Emily Scholler

**Affiliations:** Gerontological Society of America, Gerontological Society of America, District of Columbia, United States; American Academy of Family Physicians, Leawood, Kansas, United States; CEOi, Wayne, Pennsylvania, United States; High Lantern Group, Wayne, Pennsylvania, United States

## Abstract

Clinical research demonstrates the value of early detection of dementia, including dementia caused by Alzheimer’s disease (AD) and related disorders. Yet, timely and accurate diagnosis of AD in clinical practice remains challenging. The most widely used biomarkers to aid diagnosis of AD are positron emission tomography (PET) scans and cerebrospinal fluid (CSF) biomarkers. However, the clinical community recognizes that these tests have some limitations. Blood tests, also known as blood-based biomarkers (BBMs), are emerging as an aid for detecting and diagnosing AD. These include tests that measure circulating levels of amyloid or phosphorylated tau protein. The Global CEO Initiative on Alzheimer’s Disease (CEOi) is leading a global effort to prepare for the widespread adoption of BBMs for diagnosis of AD into clinical practice. As part of this effort, CEOi commissioned the American Academy of Family Physicians and GSA to issue surveys to their members in May and June 2024 to explore current perceptions and experience with BBM testing. Members were also asked to share their thoughts on how BBMs might be used in clinical practice and what is needed to strengthen their knowledge about BBMs. Insights from compatible surveys issued by AAFP and GSA are shared along with what clinicians would need to employ BBM tests in their practices.

